# Mind-mindedness in mothers of infants with excessive crying/sleeping/eating disorders

**DOI:** 10.3389/frcha.2024.1331016

**Published:** 2024-05-23

**Authors:** Maria Licata-Dandel, Susanne Kristen-Antonow, Sarah Marx, Volker Mall

**Affiliations:** ^1^ Social Pediatrics, TUM School of Medicine, Technical University of Munich, Munich, Germany; ^2^ kbo-Kinderzentrum, Munich, Germany; ^3^ Department of Psychology, Charlotte-Fresenius-University, Munich, Germany; ^4^ Developmental Psychology, Department of Psychology, Ludwig Maximilian University, Munich, Germany; ^5^ German Center for Child and Youth Health (DZKJ), Munich, Germany

**Keywords:** maternal mind-mindedness, infancy, excessive crying, sleeping disorders, eating disorders, regulatory problems/disorders

## Abstract

**Introduction:**

Excessive crying, sleeping, and eating disorders are among the most prevalent mental health diagnoses in the first 3 years of life and involve significant health service use. Parents of infants with excessive crying/sleeping/eating disorders report high levels of stress, since they feel incapable of soothing and/or nurturing their baby. Infants' distress can lead to a breakdown in parents' mentalizing abilities and, more specifically, parental mind-mindedness in the parent-child interaction. Moreover, the signals of infants with excessive crying/sleeping/eating disorders tend to be equivocal and difficult to read. This also might contribute to lower parent-child interaction quality. Until now, parental mind-mindedness, which is regarded as a prerequisite for sensitivity, has not been investigated in mothers of infants with excessive crying/sleeping/eating disorders. We investigated whether mind-mindedness in mothers of infants with excessive crying, sleeping and/or eating disorders differed from a healthy control group. We supposed that mothers of infants with excessive crying/sleeping/eating disorders would use (1) less appropriate mind-related comments (AMRCs), and (2) more non-attuned mind-related comments (NAMRCs) than mothers in the control group.

**Methods:**

Our sample consisted of 44 mothers and their infants who were patients in a socio-paediatric clinic in Germany. The children were diagnosed with excessive crying, sleeping and/or eating disorders according to DC:0-5 (= clinical group). The control group was composed of 64 healthy children and their mothers. Maternal mind-mindedness was coded during a free-play interaction.

**Results:**

Results showed that mothers of infants with excessive crying, sleeping and/or eating disorders used both more AMRCs (*p* = .029) as well as more NAMRCs (*p* = .006) than mothers in the control group.

**Discussion:**

The findings are discussed in terms of implications for interventions (e.g., enhancing mind-mindedness trough video-feedback).

## Introduction

Excessive crying, sleeping, and eating problems/disorders are highly prevalent in infancy and toddlerhood, and are one of the most frequent reasons for clinical referral (1). This symptom group is often referred to as “regulatory problems” (RPs) (2, 3). The prevalence rates vary depending on the definition of the problem (vs. disorder), the population as well as the applied measurements. With regard to excessive crying, prevalence rates vary from 5%–26% (4–6), whereas sleeping problems range from 10%–33% (7–9), and eating problems from 15%–43% (4, 9–11). About 20% of the infants show one or more (and in parts persistent) problems in those areas (2, 3, 12, 13). Infants with multiple and/or persistent RPs have a heightened risk of developing mental health problems in childhood (12, 13, 14–18), in adolescence (14), and adulthood (12, 19–22). A meta-analysis (23) showed that about 23.3% of the infants with RPs develop behavior problems during childhood, and that behavior problems are particularly pronounced in infants with multiple RPs.

Theoretical models argue that persistent RPs or clinically relevant disorders such as sleep onset or night waking disorder or excessive crying disorder (24) are best described and understood in a relational context: They are frequently embedded in a family system with high psychosocial stress and problems in the parent-child relationship/interaction (2). Indeed, studies showed that having an infant suffering from RPs puts parents under enormous stress (9, 25). Moreover, parents of infants with RPs report lower levels of self-efficacy, more social isolation (2, 26, 27) and more symptoms of depression and anxiety (28, 29). Furthermore, the parent-child interaction quality in parents of infants with RPs can be constrained (30, 31–33). Additionally, bonding difficulties (34, 35) as well as lower parental mentalizing abilities (36) in parents of infants with RPs are reported.

Maternal mind-mindedness (MM) is regarded as a “bridge” between mentalization and sensitivity “at the interface between representational and behavioral operationalization of caregiver-child interaction” [(37), p. 408] and is defined as the caregiver's proclivity to view the infant as an intentional agent with autonomous mental states. In infancy, MM is assessed using a free-play interaction between the caregiver and the infant, coding the caregiver's comments on the infant's mental states as appropriate vs. non-attuned. Appropriate mind-related comments (AMRCs) refer to the caregiver's correct interpretation of the infant's mental state, whereas non-attuned mind-related comments (NAMRCs) refer to incorrect interpretations. A large number of AMRCs (controlling for overall verbosity) is interpreted as high MM. A large number of NAMRCs is regarded as being less optimal with regard to MM, as it is indicative that caregivers misinterpret the child and are more focused on their own agenda than on the child [e.g., (38)]. AMRCs and NAMRCs are not related to each other (39–42), and are thus regarded as independent scales of the same construct [see also (43)] Beyond infancy, MM is assessed by coding the number of mental attributes the parent uses when asked to describe the child (= interview-measure).

Many studies have demonstrated that the number of the caregiver's AMRCs in the parent-child interaction is predictive of several positive outcomes on the child's side. Examples here are higher attachment security (44–48), better emotion regulation (49, 50), better executive functions (51), higher empathy (52), better theory of mind skills (53–57), and lower rates of behavior problems (58, 59). In contrast, NAMRCs have been identified as a risk factor for the development of behavior problems (60). With regard to attachment security, Meins and colleagues (41)found that both AMCRs and NAMCRs uniquely predicted child attachment security (disorganized vs. organized attachment status). Interestingly, mothers of children with an insecure-resistant attachment style produced more NAMRCs (but not more AMRCs) than mothers of insecure-avoidant children.

Studies with populations in which psychosocial risk factors are present showed heterogenous findings. In an investigation in which mothers had a severe mental illness, Schacht et al. assessed MM and found that mothers with a borderline personality disorder showed lower MM as assessed via the interview measure (i.e., they used proportionally less mental attributes to describe their child) than healthy mothers (61). In a later study, assessing AMRCs and NAMCRs in the mother-child interaction, mentally ill mothers scored lower than healthy mothers for both AMRCs and NAMRCs (62), whereas in another sample, mothers with mental illness only produced more NAMRCs than a healthy control group (62). Investigating MM (using the interview-measure) in a group of parents of children who had been referred to Child Mental Health Service, lower MM (i.e., a lower proportion of mental attributes to describe their child) was found (63), whereas in samples of parents of children with developmental disorders, no differences in MM (also using the interview-measure) were found (64, 65).

Being mind-minded towards an infant diagnosed with a mental health disorder referring to the symptom group of RPs (i.e., crying, sleeping and/or eating disorders) can be very challenging for parents, since signals of those children tend to be equivocal and difficult to read. Also, infants with RPs tend to show more difficult temperamental features, such as fussiness (66) and negative emotionality (28). Moreover, it is well known that high stress, which is common in parents of infants with RPs (2, 25), is likely to lead to a break-down of mentalizing abilities (67). Caregivers of infants with RPs might also be inconsistently attuned to infants, comparable to caregivers of children with a resistant attachment style (41). Since parenting stress (65, 68) as well as infant negative affect (69, 70) have been identified as risk factor of low MM, we supposed that maternal MM would be constrained in mothers of infants with excessive crying, eating and/or sleeping disorders. Specifically, we supposed that mothers of infants with excessive crying/sleeping/eating disorders would use (1) fewer AMRCs, and (2) more NAMRCs compared to a non-clinical control group. Moreover, as further exploratory analyses, we tested the association between maternal MM and the number of infant disorders in the clinical group.

## Materials and methods

### Study design and sample

The sample of the present study consisted of *N* = 108 children and their mothers. The clinical sample was a sub-sample (*n* = 44) that was drawn from an ongoing study that investigated psychosocial and genetic factors influencing early child mental disorders. Children were inpatients at a socio-paediatric clinic in Germany that is specialized in the treatment of early regulatory and developmental disorders. Child mental disorders were classified according to axis I of the DC:0-5 (24). The DC: 0-5 is a multiaxial classification system for children aged 0–5 years, and was developed as an attempt to do justice to the complexity of mental disorders in infancy and early childhood The clinical disorders on axis I are complemented by four other axes: relationship context (axis II), physical health and illnesses (axis III), psychosocial stressors (axis IV) and developmental skills (axis V). In the present study, only axis I diagnosis was assessed.

Inclusion criteria for the present study were as follows: (1) Children were 0–18 months old, (2) mothers had good German language competencies and spoke German with their child, (3) children were diagnosed with one or more of the following diagnoses according to DC:0-5: excessive crying disorder, sleeping disorder, eating disorder. A night waking disorder was diagnosed in 30% of the children in the clinical group. On the other hand, 26.67% suffered from a sleep onset disorder. Undereating disorders were diagnosed in 15% of the children, 6.67% fulfilled the criteria of an excessive crying disorder, and 21.87% of the children were diagnosed with “other eating/sleeping/crying disorder”. Of the children in the clinical group, 65.9% had one diagnosis, while 34.1% had multiple (more than one) diagnoses. None of the children had a global developmental delay. The mean week of gestation was *M* = 37.23 (*SD* = 5.30). 9 children were born before 37 weeks of gestation. There were no significant differences between children born before and after 37 weeks of gestation in MM within the clinical group [AMRC (t(41) = 0.23, *p* = .823, NAMRC (t(41) = −1.44, *p* = .158].

The control group (*n* = 64) were participants of a longitudinal study (2007–2017) that investigated social-cognitive development from infancy to childhood [e.g., see (71–73)]. The third measurement point of the longitudinal study (2008) was conducted when children were 12 months old. The results recorded during this measurement point were used in the present study. The families were recruited from public birth records and were mainly from the middle class of an urban area in Germany. A screening for mental health disorders was not conducted with the control group; however, parents were asked to report whether their child had any diagnosed physical or mental disorder, and whether the child received any medical care or therapy. Infants were only included if they were healthy, full-term and did not have any pre- or perinatal complications. Addresses were obtained through local birth records.

The ethics committee of the Technical University of Munich approved of the study (registration number: 2019-34_9-S-SR).

### Measures

#### Maternal MM

Maternal MM (74) was assessed using a videotaped free play interaction in a laboratory setting (control group), respectively in the clinic at the beginning of the hospital stay (clinical group). The duration of the play interaction was 9 min in the control group and *M* = 12.37 min (SD = 3.27) in the clinical group. The mothers and their child were seated on a carpet on the floor where age-appropriate toys were provided (e.g., cars, dolls, plush toys, books, toy blocks). Mothers were asked to play with their child as they usually would do at home.

First, all comments that were mind-related were identified. Mind-related comments were defined as any comment that (a) uses an internal state term to comment on the infant's mental states (e.g., talking about desires, emotions, cognitions); or (b) any utterance that is meant to be a dialogue said/thought by the infant (for a more detailed description of the coding, see (39, 75). Mind-related comments were coded as appropriate if the coder agreed with the mother's reading of the infant's current internal state (e.g., the mother says “You want the ball?” while the infant is reaching towards the ball). Furthermore, if the mother clarified how to proceed after a pause in the interaction, her comment was rated as appropriate. Comments linking a current activity with similar events in the past or future were also coded as appropriate (e.g., the mother says “Do you remember our car?” while the infant is playing with a toy car).

Mind-related comments were coded as non-attuned if the mother commented upon the infant's mental state in a non-attuned manner. Five cases in which a mental state comment was coded as non-attuned were described as follows: (a) if the researcher disagreed with the mother's reading of the infant's internal state (e.g., the mother says “You are bored with the ball” while the infant is actively playing with it); (b) if the mother commented on events which were unrelated to the infant's current activity (e.g., without having talked about the grandmother before, the mother says “Do you want to visit granny tomorrow?”); (c) if the mother tried to engage the child in a new activity while the child was actively engaged in something else; (d) if the mother attributed internal states to the infant which were not implied by the infant's behavior, but appeared to be projections of her own internal states (e.g., the mother says “You think about your dad whom you love so much, don't you?”); (e) if maternal comments were made in which the referent was not quite clear, such as “You like that!” when the infant was not attending to any particular object or event.

To control for verbosity, scores for AMRCs as well as NAMRCs were expressed as a proportion of the total number of comments, i.e., all comments (= sentences) regardless whether they contained AMRCS/NAMRCs or not). All the videos were coded by one observer, and 25 percent (*n* = 27) of the videos were coded by another observer using the verbal transcripts of the first coder, but watching the videos again. Cohen's Kappa resulted an average *κ* value of.70.

MM has been shown to have sufficient construct and predictive validity. For example, the number of AMRCs was found to be positively related to both maternal sensitivity (40, 46, 76) and child attachment security (41, 44). Also, MM has been related to children's developmental outcomes: As an example, the number of AMRCs resulted as a better predictor for later child theory of mind skills than maternal sensitivity (40). Furthermore, there is evidence for temporal stability of NAMRCs (55).

### Statistical analyses

The analyses were conducted using SPSS 29. First, significant group differences between the clinical group and the control group were investigated using *t*-Tests. Intercorrelations between all study variables were conducted in the entire sample. In order to figure out whether there were differences in AMRCs and NAMRCs between the clinical group and the control group, two ANCOVAs with group as factor and child age as covariate were conducted. Since two confirmatory analyses were carried out, *p*-values of the ANCOVAs were adjusted for multiple testing using the Bonferroni-method. Effect sizes were interpretated according to Cohen (77) (*η²*_part._* *= 0.01 = small, *η²*_part._ = 0.06 = medium, *η²*_part._ = 0.14 = large effect). In the clinical group, further exploratory analyses (correlations) between MM and number of child diagnoses were conducted.

## Results

### Descriptive analyses

Firstly, the two groups were compared with regard to their demographic characteristics. Significant group differences only emerged with regard to child age (see Table 1); thus, child age was included as a covariate in further inferential analyses.

**Table 1:** Comparison of demographic characteristics between groups.

Characteristics	Clinical group (*n* = 44)	Control group (*n* = 64)	*p*
Maternal age (years), mean (*SD*)	33.04 (4.17)	33.24 (4.37)	.490
Education/qualified for university entrance, %	63.6%	75.0%	.217
Child age (months), mean (*SD*)	9.61 (4.02)	11.63 (.41)	.002**
Child gender, %	45.5%	43.8%	.739
Single child, %	68.3%	65.6%	.570

** *p* < .01, two-tailed significance level.

The descriptives of maternal MM are depicted in Table 2. The number of both AMRCs and NAMRCs were calculated as weighted scores, dividing the number of AMRCs and NAMRCs through the total number of comments.

**Table 2:** Frequencies of total comments and mind-mindedness comments, as well as frequencies of mind-mindedness comments weighted by total comments.

	Clinical group (*n* = 44)		Control group (*n* = 64)	
	Mean (SD)	Range	Mean (SD)	Range
Total Comments	81.45 (42.45)	15–203	103.36 (34.97)	18–181
AMRCs	5.18 (4.67)	1–23	4.19 (4.08)	0–17
NAMRCs	1.18 (1.60)	0–6	0.59 (1.08)	0–5
Weighted AMRCs	0.07 (0.04)	0.01–0.21	0.04 (0.03)	0.00–0.11
Weighted NAMRCs	0.02 (0.02)	0.00–0.10	0.00 (0.01)	0.00–0.03

Intercorrelations of the study variables (demographic characteristics, AMRCs and NAMRCs) were carried out using the entire sample (*N* = 108) (Table 3).

**Table 3:** Intercorrelations among the study variables (*N* = 108).

Variables	1	2	3	4	5	6	7
1. AMRCs	–						
2. NAMRCs	.09	–					
3. Maternal education	.02	.09	–				
4. Child gender	−.02	−.06	−.07	–			
5. Child age	−.45***	−.16	−.03	.09	–		
6. Maternal age	.03	−.04	−.02	.02	.06	–	
7. Single child	.01	−.06	−.05	.04	.01	−.29*	–

* *p* < .05, ***p < .001, two-tailed significance level; AMRCs and NAMRCs are weighted scores.

### Inferential analyses

**Figure 1:**
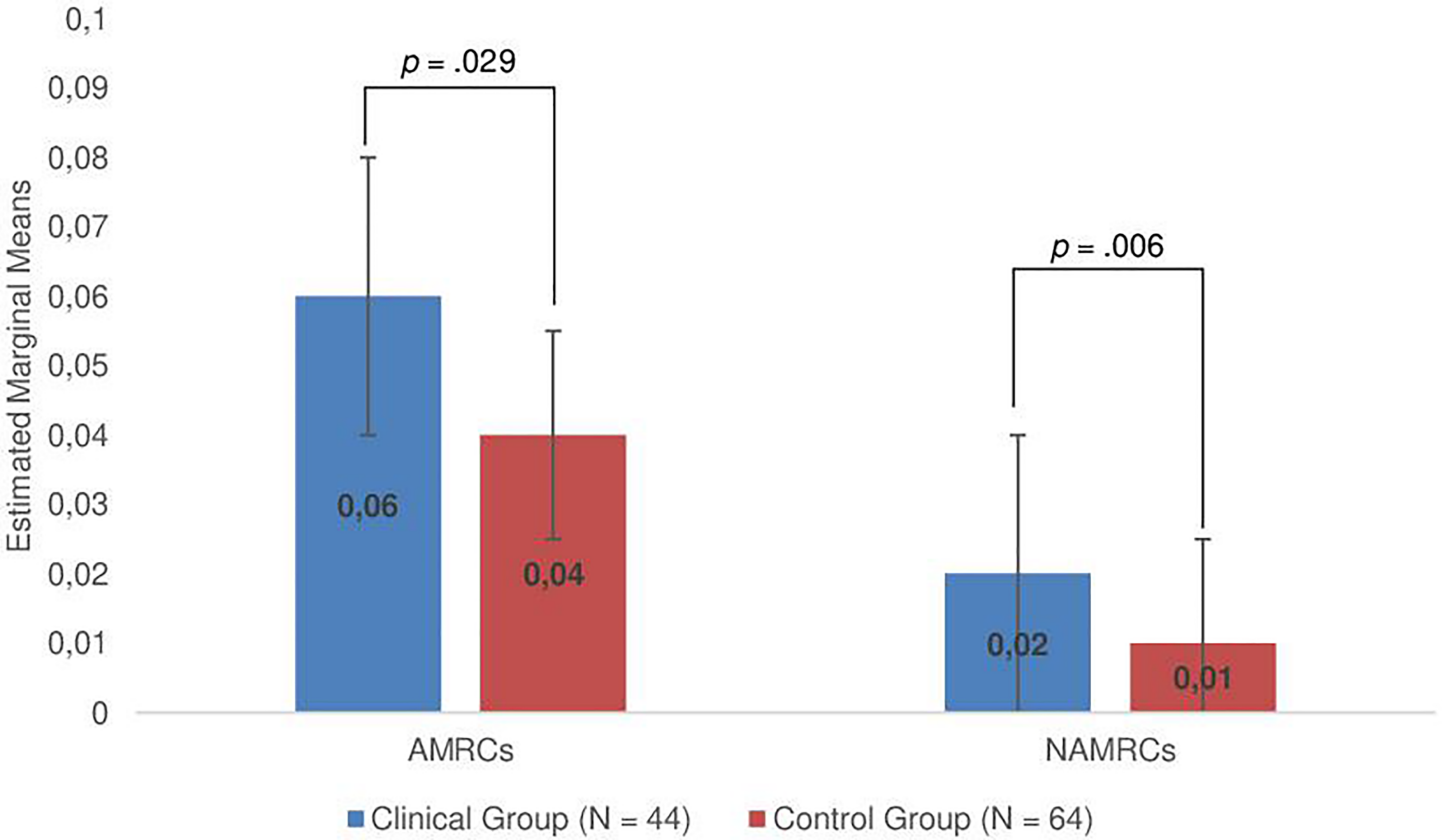
Group differences regarding maternal MM (controlling for child age; error bars show standard errors); *p < .05, **p < .01, two-tailed significance level. AMRCs (appropriate mind-related comments) and NAMRCs (non-attuned mind-related comments) are weighted scores. *P*-values were adjusted using the Bonferroni-method.

Two ANCOVAs with group (clinical/nonclinical) as factor and (1) AMRCs as well as (2) NAMRCs as dependent variables and child age as a covariate were calculated. Results showed that mothers in the clinical group produced significantly more AMRCs, *F*(1, 105) = 6.16, *p_Bonferroni_* = .029, *η*²_part._ = .06, as well as more NAMRCs, *F*(1, 105) = 9.20 *p_Bonferroni_* = .006, *η*²_part._ = .08, than the control group (see Figure 1). Given our sample size of *N* = 108, these effect sizes can achieve a power of 0.73 and 0.82 respectively.

Further exploratory analyses between maternal MM and the number of child disorders (single vs. multiple disorders) within the clinical group showed a significant positive correlation between NAMRCs and the number of child disorders, *r* = .338 (*p* = .025), whereas the number of AMRCs were not significantly related to the number of disorders (*r *= −.233, *p* = .128).

## Discussion

The present study is the first study to investigate maternal MM in a group of infants with mental health disorders and to apply the DC:0-5 in order to diagnose excessive crying, sleeping, and eating disorders (Table 4). In the study, maternal MM in mothers of infants with excessive crying, sleeping and/or eating disorders were compared to maternal MM in a nonclinical control group. We supposed that mothers in the clinical group would use less AMCRs and more NAMCRs than mothers of the control group.

**Table 4:** Frequency and percentages of diagnoses.

Diagnosis	Frequency	%
Sleep onset disorder	16	25.81
Night waking disorder	18	29.03
Undereating disorder	9	14.52
Exzessive crying	4	6.45
Other crying/sleeping/eating disorders		
Other sleeping disorders	6	9.68
Other eating disorders	8	12.90
Other crying disorders	1	1.61

Multiple selection of different diagnoses possible.

Regarding the descriptive results, correlational analyses revealed that infant age was negatively associated with AMCRs. This might indicate that with growing age, children's mental states become harder to read, likely because their theory of mind further develops (78), and when they begin to walk, their social world becomes increasingly larger (79). However, these results should not be overstated due to the exploratory character of the analyses.

Inferential analyses showed that mothers in the clinical group used both more AMRCs as well as more NAMRCs than the control group, with a moderate effect size. This result was surprising as we expected mothers to make less AMRCs instead of more AMRCs. It indicates that parents of infants with excessive crying, sleeping and/or eating disorders do not lose their interest, but rather have a high propensity to comment on their infant's mental states—which is likely to happen as a result of repeated experiences of unsuccessful attunement. In fact, they tend to use lots of mind-related comments, probably as desperate attempts to make sense of their child's equivocal signals. This is comparable to hyper-mentalizing (i.e., the tendency to excessively try to make sense of behaviors, often leading to misinterpretations of others’ mental states), which is also regarded as low reflective functioning (80). The results of this study are thus to a certain extent in line with studies reporting lower MM as well as lower reflective functioning in parents of children with mental health problems (63, 81, 82). Specifically, our findings are to a certain extent in line with Georg et al. who found more pre-mentalizing in mothers of infants with regulatory problems (36). Pre-mentalizing isn't directly comparable to the MM coding, as MM doesn't assess if mother's comments are hostile. Pre-mentalizing means that parents don't see infants’ behavior as an expression of their mental states, and thus view it as hostile vs. that they mentalize but misinterpret their child's mental states. However, what often follows from pre-mentalizing is that parents attribute the wrong intentions to the child which is comparable to some types of NAMCRs. Our results also fit to Meins et al.'s (56) finding that mothers of infants with an insecure-resistant attachment style produce more NAMRCs than mothers of infants with an insecure-avoidant attachment style: Mothers of so-called “difficult” children (i.e., children with an insecure-ambivalent attachment style and/or children with a regulatory disorder) seem to have the tendency to refer a lot to the child's mental states, but often misinterpret their child's signals. Thus, consistently taking the child's perspective seems to be much more difficult for parents having a “difficult to read”-child with a mental health disorder/regulatory disorder.

Further exploratory analyses showed that the number of child disorders was related to more NAMRCs, indicating that severity of the child's mental illness is associated with a greater probability to misinterpret the child’s mental states. It might be that parents of an infant with excessive crying or/and sleeping/eating disorders, in some instances, tend to interpret the infant's signals as being intentional and hostile against the parent since they are not successful in soothing or nurturing their baby. This experience can, in turn, lead to high stress levels, feelings of guilt, but also aggression (83, 84). In the case of high stress, reflective functioning in the context of relationships tends to break down, leading to more pre-mentalizing modes (85).

In sum, the present study supports theories on parental reflective functioning [e.g., (85)]. It shows that maternal MM can be rather inconsistent (both appropriate and non-attuned) in the context of infant mental health disorders/regulatory disorders. This could be the starting point of a vicious circle in the parent-child interaction (2, 86) and enhance the risk of negative sequel with regard to child development (13, 16). However, both directions of effects seem reasonable. Parents may be more inconsistent in MM as a result of the child's difficulties/disorder, or inconsistent MM may influence child difficulties/disorder. This unanswered question should be investigated in depth by applying a longitudinal design beginning ideally directly after childbirth.

Following up on Bilgin and Wolke's (87) findings that there were no reciprocal relations between infant regulatory problems and maternal sensitivity, it might be that the production of many AMRCs compensates for many NAMRCs. This would implicate that using many NAMRCs does not necessarily lead to lower sensitivity, given that mothers use more AMRCs at the same time. This hypothesis should be tested in subsequent studies.

### Strengths and limitations

This is one of the first studies to use the DC:0-5 for diagnosing mental health disorders in infancy. Furthermore, this is the first study using the observational measure of MM in the context of infant mental health disorders. Thus, a strength of the study is the exclusive use of objective measures, which are known to be more valid than self-report measures [e.g., (88)].

The study also has some limitations. First, mental health status was not assessed via a diagnostic tool in the control group. Thus, it cannot be ruled out completely that mental health disorders were also present in the control group. Another limitation is that both participants of the clinical as well as the control group were rather highly educated, which limits generalizability of the results. Furthermore, whereas the control group was assessed in 2008, the clinical group was recruited from 2020 to 2023. Thus, it cannot be ruled out that the assessment of the two groups in different decades might have affected our results. Even if research doesn't report any significant changes in means of MM across mothers born in different decades, the study should be replicated with mothers raising their kids in the same decade. Also, causal conclusions may not be drawn from our findings due to the cross-sectional design of the study. As mentioned above, future studies should use a longitudinal design with several measurement points in order to rule out the direction of effects.

### Implications for clinical practice

Our findings have important implications for intervention. As prior research has shown that maternal MM can be regarded as prerequisite for sensitivity (44), and is a predictor of child secure attachment (40), it is worth focusing on the improvement of MM in the treatment of excessive crying, sleeping, and eating disorders. Our results indicate that especially parents who have a child with multiple disorders need help urgently. Early intervention in the context of severe RPs targeting toward strengthening the parent-child relationship is crucial in order to prevent negative developmental pathways (89). One intervention that focuses explicitly on parental reflective functioning is “Minding the Baby” [MTB, (82)]. MTB is an attachment-based, interdisciplinary home visiting intervention, aiming at the promotion of parental reflective functioning from pregnancy onward. It includes integrating elements of infant–parent psychotherapy, adult psychotherapy, family/couple counseling as well as concrete support of the parent. Slade et al. demonstrated the effectiveness of the intervention (82). The authors showed that reflective functioning was more likely to increase over the course of the intervention in mothers of the intervention group compared to mothers of the control group, and that infants of the intervention group were more likely to show secure attachment. Furthermore, video intervention therapy [VIT, (90)] is a well-established and widely used intervention in the treatment of infant regulatory disorders [see also (3)]. Thus, a combination of a mentalization based approach together with interventions targeting at enhancing maternal sensitivity also on a behavioral level (e.g., through video feedback) might be a very helpful approach in the context of early regulatory disorders. Further research is needed to evaluate the effectiveness of concrete intervention strategies in the context of early regulatory disorders.

## Data Availability

The raw data supporting the conclusions of this article will be made available by the authors, without undue reservation.
